# N-terminal pro-brain natriuretic peptide levels associated with severe hand, foot and mouth disease

**DOI:** 10.1186/s12879-016-1929-9

**Published:** 2016-10-19

**Authors:** Hui-Ling Deng, Yu-Feng Zhang, Ya-Ping Li, Yu Zhang, Yan Xie, Jun Wang, Xiao-Yan Wang, Shuang-Suo Dang

**Affiliations:** 1 Department of Infectious Diseases, Second Affiliated Hospital of Medical College of Xi’an Jiaotong University, Xi’an, 710004 China; 2 Department of Infectious Diseases, Xi’an Children’s Hospital, Xi’an, 710003 China

**Keywords:** Hand, foot, and mouth disease, N-terminal pro-brain natriuretic peptide, Disease severity, Mortality

## Abstract

**Background:**

Severe hand, foot, and mouth disease (HFMD) is sometimes associated with serious complications such as acute heart failure that can cause substantial child mortality. N-terminal pro-brain natriuretic peptide (NT-proBNP) is a sensitive and specific biomarker of congestive heart failure. The aim of this study was to use plasma NT-proBNP levels to establish the severity of childhood HFMD.

**Methods:**

A retrospective study was performed in 128 Chinese patients with severe HFMD and 88 patients with mild HFMD treated between January 2014 and October 2015. Univariate and multiple logistic regression analyses were used to analyze the risk factors for severe HFMD. NT-proBNP levels were analyzed in 128 severe HFMD patients, and the predictive value of NT-proBNP was assessed by receiver operating characteristic analyses.

**Results:**

Multivariate analysis controlling for several potential confounders showed that enterovirus 71 infection [odds ratio (OR) 19.944, 95 % confidence interval (CI) 6.492–61.271], peripheral WBC count (OR 3.428, 95 % CI 1.186–9.914), fasting glucose (OR 19.428, 95 % CI 2.236–168.784), procalcitonin (OR 9.084, 95 % CI 3.462–23.837, and NT-proBNP (>125 pg/mL) (OR 16.649, 95 % CI 4.731–58.585) were each associated with the severity of HFMD. The 45 dead severe patients had higher pre-procedural levels of NT-proBNP than the 83 cured severe patients (12776 ± 13115 versus 1435 ± 4201 pg/mL, *P* < 0.001). An NT-proBNP cutoff value of 982 pg/mL predicted mortality with 87 % sensitivity and 86 % specificity.

**Conclusion:**

Plasma NT-pro-BNP level appears to be a useful biological marker for predicting the severity and mortality of HFMD.

**Electronic supplementary material:**

The online version of this article (doi:10.1186/s12879-016-1929-9) contains supplementary material, which is available to authorized users.

## What is Known

• *Severe HFMD cases and over 90 % of fatal cases were caused by EV71. Acute heart failure is one of the most common causes of death in severe HFMD*. N-terminal pro-brain natriuretic peptide (NT-proBNP) *are useful biomarkers for the assessment of congestive heart failure.*

## What is New

• *EV71 infection, fasting glucose, procalcitonin (PCT) and NT-proBNP levels were each associated with the severity of HFMD*. Plasma NT-pro-BNP level appears to be a useful biological marker for predicting the severity and mortality of HFMD.

## Background

Severe hand, foot and mouth disease (HFMD) associated with enterovirus (EV) 71 in children can result in high morbidity and mortality [1–3]. Most children with HFMD have mild symptoms including fever and cutaneous lesions on their hands, feet and buttocks, along with oral lesions [4, 5]. However, in rare cases, patients may also develop severe and life-threatening complications such as encephalitis, acute pulmonary edema, and cardiopulmonary failure [2, 6]. Consequently, acute heart and pulmonary failure is proposed as an important cause of rapid deterioration of HFMD, leading to mortality [3, 7].

Since 2008, nationwide epidemics of EV71 have occurred [8–10] in China, affecting 4.5 million children, and >3500 have died from the disease due to neurogenic cardiopulmonary failure and brainstem encephalitis, according to the Chinese Center for Disease Control and Prevention (http://www.chinacdc.cn/). These outbreaks of HFMD and high mortality represent a significant public health problem in China. However, there is still no available vaccine against EV71 and other enteroviruses that cause HFMD [11–13]. Current management in the clinic is only to relieve symptoms. Actually, it is important and arduous to predict whether patients will develop severe or life-threatening illness at their first clinical visit. Severe HFMD progresses quickly and has high mortality. Feasible prognosis prediction of severe HFMD would aid clinical decisions and management.

The 108-amino-acid (aa) pro-hormone brain-type natriuretic peptide (BNP) was first isolated from porcine brains in 1988 [14, 15]. BNP is a cardiac neuro-hormone secreted from ventricular myocytes in response to increased intraventricular pressure [15–17]. Once BNP has been segregated, it divides into a biologically active 32-aa BNP and inactive 76-aa NT-proBNP [18, 19]. Excessive volume or pressure loading of the heart can lead to increased BNP synthesis and secretion. In clinical applications, BNP levels are related to cardiac function and are thought to be a sensitive and specific biomarker of congestive heart failure [16, 19, 20]. Some studies have established that BNP is a risk marker in patients with HIV infection [21], severe sepsis [18], acute Kawasaki disease [22], and acute Puumala hantavirus infection [23]. A recent HFMD investigation has demonstrated that high levels of NT-proBNP are associated with the complication of cardiopulmonary collapse [24].

In this study, we explored the hypothesis that higher levels of NT-proBNP might be associated with severe HFMD.

## Methods

### Patients

This study was approved by the Medical Ethics Committee of the Second Affiliated Hospital of the Medical College of Xi’an Jiaotong University and Xi’an Children’s Hospital, Xi’an, China. A total of 2320 HFMD patients were collected from all cases admitted to the two hospitals between January 2014 and October 2015. We retrospectively enrolled 216 patients whose clinical data were elaborate, comprising 128 Chinese patients with severe HFMD and 88 patients with mild HFMD in this case–control study. Among the 128 patients with severe HFMD, 45 died. Children with severe HFMD were treated as cases, while those with mild disease served as controls and were used as the reference group when calculating odds ratios (ORs).

The definition of HFMD was based on the criteria in the Hand, Foot and Mouth Disease Clinical Guide (2010 edition) [25], which contains the following criteria: (1) symptoms and signs occur during epidemics in preschool children; and (2) patients show typical exanthema on the hands, feet, mouth and/or buttocks, with or without fever. Patients with mild HFMD had rashes on their hands, feet, mouths, and buttocks, with or without fever. Patients with severe disease may have one of the followings: (1) neurological manifestations; (2) respiratory manifestations; and (3) circulatory manifestations. The diagnosis of fatal HFMD was based on one of the following criteria: (1) frequent convulsions, coma and cerebral hernia; (2) dyspnea, cyanosis, bloody frothy sputum, and pulmonary rales; and (3) shock and circulatory failure. Other concomitant viruses positive for IgM were ruled out in this study.

### Pan-enterovirus RT-PCR assay for identification of EV71 infection

EV71 infection in patients with clinically suspected HFMD was confirmed by positive EV71 identification using pan-enterovirus real-time RT-PCR. Viral RNA extraction from throat swabs was performed using an RNA extraction kit (Qiagen, Germany). Real-time RT-PCR for enterovirus was performed with the primers 5′-TCCTCCGGCCCCTGAATG -3′ and 5′-AATTGTCACCATAAGCAGCCA-3′. This triplex RT-PCR reaction was performed in a 25 μL total reaction mixture containing 17 μL buffer, 1.5 μL primer and probe mix, 1.5 μL enzyme mix, and 5 μL extracted RNA template. On a CFX96 Real-Time PCR Detection System (Bio-Rad Inc., USA) 30 min reverse transcription at 50 °C and 10 min denaturation at 95 °C, followed by 5 circles of pro-amplification at 94 °C for 10 s, 50 °C for 30 s, 72 °C for 30 s, and 40 cycles at 94 °C for 10 s, and 58 °C for 30 s were performed to collect fluorescence signal.

### Plasma NT-proBNP measurement

A rapid, comer cially available NT-proBNP immunoassay (Cobas, Germany) was used for the NT-proBNP measurement. Blood samples were collected by venipuncture into standard EDTA tubes and centrifuged within 30 min of collection. Plasma NT-proBNP was measured using a commercially available horse-radish peroxidase, and colorimetric end-point assay was carried out for the quantitative determination of feline NT-proBNP.

### Data collection

The authors (Deng HL and Zhang YF) used standardized forms to extract data independently from medical records. Detailed demographic data including sex and age were collected from medical records. Clinical manifestations including highest fever temperature, vomiting, limb weakness, hypersomnia, convulsion, high blood pressure, consciousness disorder, dysfunction of respiratory rhythm, and circulatory disturbance were collected and 128 severe cases were identified. The laboratory data collected included enterovirus infection, white blood cell (WBC) count, fasting glucose, plasma NT-proBNP levels, myocardial enzyme spectrum [creatine kinase (CK), CK-MB, lactate dehydrogenase (LDH)] and procalcitonin (PCT).

### Statistical analysis

All statistical analyses were performed using SPSS version 13.0 (IBM, Chicago, IL, USA). Data for continuous variables were summarized as mean ± SD or median (range), and inter-group differences were assessed for significance using the Wilcoxon rank-sum test or Student’s *t* test. Data for categorical variables were summarized as numbers and percentages, and the χ^2^ test was used to assess differences between patients with mild or severe HFMD. Univariate and multivariate logistic regression analyses were used to identify risk factors associated with severe HFMD using ORs. All variables with a univariate *P* < 0.20 along with those deemed to be clinically significant were considered for inclusion in multivariate models. Receiver operating characteristic (ROC) method was used to define the optimal cut-off values of baseline NT-proBNP. The threshold of significance for all statistical tests was defined to be *P* < 0.05.

## Results

### Patient characteristics and clinical manifestations

There were 216 patients diagnosed with HFMD including 128 severe cases, 45 of whom died (20.8 %). Table 1 shows the clinical manifestations of the 216 children with HFMD who were eligible for enrollment. There were significant differences between mild and severe cases in terms of fever, peak temperature, duration of fever, symptoms of central nervous and cardiopulmonary systems, WBC count (>15 × 10^9^/L), fasting glucose (>8.3 mmol/L), current EV71 infection, CK (>229 U/L), CK-MB (>25 U/L) and LDH (>450 U/L), PCT (>0.1 ng/mL) and plasma NT-proBNP (log_10_ pg/mL) (2.18 ± 0.35 vs 3.07 ± 0.75, *P* < 0.001) levels. Pan-enterovirus real-time RT-PCR was performed to confirm the cases caused by EV71 infection. Additional file 1: Table S1 shows the clinical characteristics of HFMD caused by EV71. The proportion of severe cases among children with HFMD caused by the EV71 infection increased significantly than non-EV71 infection (*P* < 0.05). The plasma NT-proBNP (log_10_ pg/mL) levels in the EV71 infection cases were significantly higher than non-EV71 infection (3.04 ± 0.88 vs 2.49 ± 0.58, *P* < 0.001).

**Table 1:** Risk factors for severe HFMD

Relevant factors	Mild group *n* = 88 (%)	Severe group *n* = 128 (%)	*P*
Gender			0.817
Male	55 (62.5)	78 (60.9)	
Female	33 (37.5)	50 (39.1)	
Age (years)			0.557
≤ 3	65 (73.9)	99 (77.3)	
3–6	23 (26.1)	29 (22.7)	
Fever	81 (92.0)	128 (100.0)	0.001**
Temperature (°C)			0.000***
37.3 to ≤38	8 (9.1)	0 (0)	
38–39	38 (43.2)	21 (16.4)	
39–40	42 (47.7)	107 (83.6)	
Duration of fever (day)			
≤ 3	65 (73.9)	39 (30.5)	0.000***
> 3	23 (26.1)	89 (69.5)	
Hypersomnia	2 (2.3)	118 (92.2)	0.000***
Hyperarousal	38 (43.2)	121 (94.5)	0.000***
Limb shaking	5 (5.7)	98 (76.6)	0.000***
Convulsion	2 (2.3)	33 (25.8)	0.000***
Vomiting	16 (18.2)	87 (68.0)	0.000***
Dyspnoea	0 (0)	71 (55.5)	-
Pathologic reflexes	0 (0)	128 (100.0)	-
Consciousness disorder	0 (0)	75 (58.6)	-
Increased blood pressure	0 (0)	65 (50.8)	-
Circulatory disturbance	0 (0)	75 (58.6)	-
Laboratory examination			
Peripheral WBC count > 15 × 10^9^/L	12 (13.6)	56 (43.8)	0.000***
Fasting blood glucose level > 8.3 mmol/L	1 (1.1)	62 (48.4)	0.000***
EV71-positivity	11 (12.5)	75 (58.6)	0.000***
NT-proBNP (log_10_ pg/mL)	2.18 ± 0.35	3.07 ± 0.75	0.000***
NT-proBNP >125 pg/mL	49 (55.7)	120 (93.8)	0.000***
Increased CK	3 (3.4)	31 (24.2)	0.000***
Increased CK-MB	36 (40.9)	58 (45.3)	0.521
Increased LDH	2 (2.3)	18 (14.1 %)	0.001**
Increased PCT	22 (25.0)	71 (55.5 %)	0.000***
Death^a^	0 (0)	45 (35.2 %)	-
Treatment	Symptomatic treatment	Symptomatic treatment, glucocorticoid, IVIG, complication therapy	-

*HFMD* hand, foot, and mouth disease, *EV71* enterovirus 71, *WBC* White blood cell, *NT-proBNP* N-terminal of the prohormone brain natriuretic peptide, *CK* Creatine kinase isoenzyme, *CK-MB* Creatine kinase isoenzymeMB, *LDH* Lactate dehydrogenase, *PCT* procalcitonin, *IVIG* Intravenous immunoglobulins

^a^Causes of death were acute pulmonary edema, brainstem encephalitis and circulatory failure

*P <0.05, **P <0.01,***P <0.001

### Risk factors for severe disease

Risk factors for severe HFMD are summarized in Table 2. In the univariate analysis, current EV71 infection [(OR 9.906, 95 % confidence interval (CI) 4.807–20.413], WBC count (OR 4.926, 95 % CI 2.442–9.938), fasting glucose (OR 81.727, 95 % CI 11.045–604.760), PCT (OR 3.737, 95 % CI 2.061–6.777) and NT-proBNP levels (>125 pg/mL) (OR 11.939, 95 % CI 5.205–27.383) were risk factors for severe HFMD. In the multivariate model, EV71 infection (OR 19.944, 95 % CI 6.492–61.271), WBC count (OR 3.428, 95 % CI 1.186–9.914), fasting glucose (OR 19.428, 95 % CI 2.236–168.784), PCT (OR 9.084, 95 % CI 3.462–23.837) and NT-proBNP (>125 pg/mL) (OR 16.649, 95 % CI 4.731–58.585) were each associated with the severity of HFMD.

**Table 2:** ORs for severe HFMD

Risk factors	Unadjusted OR (95 % CI)	Adjusted^a^OR (95 % CI)
Gender	0.936 (0.535-1.637)	1.105 (0.442–2.759)
Age	0.828 (0.441–1.555)	2.070 (0.692–6.815)
EV71-positivity	9.906 (4.807–20.413)***	19.944 (6.492–61.271)***
Peripheral WBC count >15 × 10^9^/L	4.926 (2.442–9.938)***	3.428 (1.186–9.914)*
Fasting glucose >8.3 mmol/L	81.727 (11.045–604.760)***	19.428 (2.236–168.784)**
PCT >0.1 ng/mL	3.737 (2.061–6.777)***	9.084 (3.462–23.837)***
NT-proBNP > 125 pg/mL	11.939 (5.205–27.383)***	16.649 (4.731–58.585)***

*HFMD* hand, foot, and mouth disease, *EV71* enterovirus 71, *PCT* procalcitonin, *NT-proBNP* N-terminal of the prohormone brain natriuretic peptide

*CI* Confidence interval, *OR* odds ratio. OR was calculated using the mild children as a reference group

^a^In multivariate logistic regression model (*n* = 216), we controlled for age, gender, EV71-seropositivity, WBC, fasting glucose, CK, LDH, PCT and NT-proBNP. After adjusting for potential confounding factors, there was significant difference in EV71 infection, peripheral WBC count, fasting blood glucose, PCT and plasma NT-proBNP levels

*P <0.05, **P <0.01, ***P <0.001

### NT-proBNP levels in severe HFMD

**Fig. 1:**
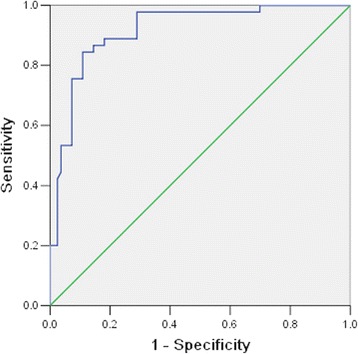
ROC curve and interactive dot diagram for calculating optimal cut-off value of NT-proBNP in predicting mortality

Children with severe HFMD were divided into two groups based on cure or death. Table 3 shows that hypersomnia, convulsion, peripheral WBC count, fasting blood glucose level, NT-proBNP levels, CK and LDH were associated with mortality in children with severe HFMD (*P* < 0.05). The ROC curve and interactive dot diagram for calculating the optimal cut-off value of NT-proBNP in predicting mortality is shown in Fig. 1. At an NT-proBNP cut-off value of >982.45 pg/mL, the sensitivity and specificity were 86.7 and 85.5 %, respectively.

**Table 3:** Risk factors associated with death of children with severe HFMD

Relevant factors	Cure group *n* = 83 (%)	Death group *n* = 45 (%)	*P*
Gender (male/female)	51/32	27/18	0.873
Age (≤3 years)	60 (72.3)	39 (86.7)	0.064
Fever	83 (100.0)	45 (100.0)	-
Peak temperature (>39 °C)	69 (83.1)	38 (84.4)	0.848
Hypersomnia	73 (88.0)	45 (100.0)	0.015*
Hyperarousal	79 (95.2)	42 (93.3)	0.661
Limb shaking	60 (72.3)	38 (84.4)	0.121
Convulsion	27 (32.5)	6 (13.3)	0.018
Vomiting	56 (67.5)	31 (68.9)	0.870
Dyspnoea	30 (36.1)	45 (100.0)	-
Pathologic reflexes	83 (100.0)	45 (100.0)	-
Consciousness disorder	30 (36.1)	45 (100.0)	-
Increased blood pressure	20 (24.1)	45 (100.0)	-
Circulatory disturbance	30 (36.1)	45 (100.0)	-
Laboratory examination			
Peripheral WBC count > 15 × 10^9^/L	27 (32.5)	29 (64.4)	0.001**
Fasting blood glucose level > 8.3 mmol/L	25 (30.1)	37 (21.8)	0.000***
EV71-positivity	43 (51.8)	32 (71.1)	0.000***
NT-proBNP (pg/mL)	1435 ± 4201	12776 ± 13115	0.000***
NT-proBNP (log_10_ pg/mL)	2.68 ± 0.49	3.79 ± 0.61	0.000***
Increased CK	13 (15.7)	18 (40.0)	0.002**
Increased CK-MB	33 (39.8)	25 (55.6)	0.087
Increased LDH	4 (4.8)	14 (31.1)	0.000***
Increased PCT	48 (57.8)	23 (51.1)	0.465

*HFMD* hand, foot, and mouth disease, *EV71* enterovirus 71, *WBC* White blood cell, *NT-proBNP* N-terminal of the prohormone brain natriuretic peptide, *CK* Creatine kinase isoenzyme, *CK-MB* Creatine kinase isoenzyme-MB, *LDH* Lactate dehydrogenase, *PCT* procalcitonin, *IVIG* Intravenous immunoglobulins

*P <0.05, **P <0.01,***P <0.001

## Discussion

We found that current EV71 infection, peripheral WBC count, fasting glucose, PCT and NT-proBNP levels (>125 pg/mL) were each associated with severity of HFMD. Consequently, ROC curve analysis revealed that an NT-proBNP value of ≥982.45 pg/mL had a sensitivity of 86.7 % and specificity of 85.5 % in predicting death in patients with severe HFMD. Therefore, early identification of these risk factors and timely and effective intervention are important in controlling mortality of severe HFMD.

Serum BNP is mainly synthesized and secreted by ventricular myocytes, and increased intraventricular pressure stress could modulate synthesis of BNP [15–17]. In addition to hemodynamic stress, inflammation of the myocardial tissue may also induce the production of BNP [22, 26]. As a clinically valuable biomarker, NT-proBNP can fulfill most of these criteria in patients with heart failure and ventricular overload [27]. In the cardiology literature, NT-proBNP had emerged as an independent and crucial warning factor of clinical outcome in patients with heart failure [19, 20]. We speculated that fatal HFMD involves brainstem and autonomic nerve dysfunction, leading to a “sympathetic storm” and significantly increased catecholamine concentration in the blood [28]. This may lead to increased blood flow to the heart and ventricular preload, resulting in increased NT-proBNP secretion and release. Previous studies have reported that NT-proBNP levels are significantly increased in severe HFMD, with cardiopulmonary collapse [24] or EV71 infection [16], which is consistent with our findings that children with severe HFMD, and those who have died, had an increase in the levels of NT-proBNP.

EV71 is a neurotropic virus that can cause severe complications involving the central neurogenic pulmonary edema, aseptic meningitis, brainstem encephalitis, and cardiopulmonary failure [6]. Many clinical studies have shown that the symptoms of HFMD caused by EV71 are more severe than those caused by other enteroviruses [29, 30]. In this study, we found that EV71 infection was associated with severe HFMD, which is consistent with previous studies. Peripheral WBC count, increased fasting glucose and PCT levels were other risk factors identified in our study, which is also consistent with other studies. PCT is a helpful biomarker for early diagnosis of inflammatory reactions, and high plasma PCT concentrations were associated with severe fatal HFMD. Increased fasting glucose was a significant risk factor in fatal cases, which is consistent with previous studies. The specific mechanism of increased fasting glucose is still unclear.

This study had some limitations. The first was that the cases of HFMD in this study were inpatients. We excluded outpatients because of incomplete medical records, and this may have introduced selection bias. The second was the small sample size in our study, and this may have led to negative results for some analyzed factors. Further studies with larger numbers of cases are required to confirm these results.

## Conclusions

EV71 infection, peripheral WBC count, fasting glucose, PCT and NT-proBNP levels (>125 pg/mL) were each associated with the severity of HFMD. Plasma NT-pro-BNP level appears to be a useful biological marker for predicting the severity and mortality of HFMD. Further studies are needed to confirm our findings.

## Additional file

Additional file 1: Table S1. The characteristics of HFMD causing by EV71. (DOC 57 kb)

## Abbreviations

BNP: Brain-type natriuretic peptide
CI: Confidence interval
CK: Creatine kinase
CK-MB: Creatine kinase isoenzyme-MB
EV71: Enterovirus 71
HFMD: Hand foot, and mouth disease
LDH: Lactate dehydrogenase
NT-proBNP: N-terminal of the prohormone brain natriuretic peptide
OR: Odds ratio
PCT: Procalcitonin
WBC: White blood cell
