# Supplementary material for: N-terminal pro-brain natriuretic peptide levels associated with severe hand, foot and mouth disease
Source: BMC Infect Dis. 2016 Oct 19;16:585. doi: 10.1186/s12879-016-1929-9 (PMC5069980; doi:10.1186/s12879-016-1929-9)
Supplement: Additional file 1: Table S1. — The characteristics of HFMD causing by EV71. (DOC 57 kb) [file 12879_2016_1929_MOESM1_ESM.doc]

**Additional table 1** The characteristics of HFMD causing by EV71

| Characteristics | EV71 positive group  *n*=86 (%) | EV71 negative group  *n*=130 (%) | *P* |
| --- | --- | --- | --- |
| Severity classification |  |  | 0.000*** |
| Mild | 11 (12.8) | 77 (59.2) |  |
| Severe | 75 (87.2) | 53 (40.8) |  |
| Gender |  |  | 0.384 |
| Male | 56 (65.1) | 77 (59.2) |  |
| Female | 30 (34.9) | 53 (40.8) |  |
| Age (years) |  |  | 0.580 |
| ≤3 | 67 (77.9) | 97 (74.6) |  |
| 3-6 | 19 (22.1) | 29 (25.4) |  |
| Fever | 84 (97.7) | 125 (96.2) | 0.537 |
| Temperature (℃) |  |  | 0.005** |
| 37.3 to ≤38 | 2 (2.3) | 6 (4.6) |  |
| 38 -39 | 14 (16.3) | 45 (34.6) |  |
| 39-40 | 70 (81.4) | 79 (60.8) |  |
| Duration of fever (day) |  |  |  |
| ≤3 | 26 (30.2) | 78 (60.0) | 0.000*** |
| >3 | 60 (69.8) | 52 (40.0) |  |
| Hypersomnia | 72 (83.7) | 48 (36.9) | 0.000*** |
| Hyperarousal | 79 (91.9) | 80 (61.5) | 0.000*** |
| Limb shaking | 61 (70.9) | 42 (32.3) | 0.000*** |
| Convulsion | 17 (19.8) | 18 (13.8) | 0.248 |
| Vomiting | 51 (59.3) | 52 (40.0) | 0.005** |
| [Dyspnoea](http://dict.cn/dyspnoea) | 65 (75.6) | 6 (4.62) | 0.000*** |
| Pathologic reflexes | 75 (87.2) | 53 (40.8) | 0.000*** |
| Consciousness disorder | 70 (81.4) | 5 (3.85) | 0.000*** |
| Increased blood pressure | 60 (72.1) | 5 (3.85) | 0.000*** |
| Circulatory disturbance | 70 (81.4) | 5 (3.85) | 0.000*** |
| Laboratory examination |  |  |  |
| Peripheral WBC count > 15×10^9^/L | 32 (37.2) | 36 (27.2) | 0.140 |
| Fasting blood glucose level > 8.3 mmol/L | 45 (52.3) | 18 (13.8) | 0.000*** |
| NT-proBNP (log_10_ pg/mL) | 3.04 ± 0.88 | 2.49 ± 0.58 | 0.000*** |
| NT-proBNP >125 pg/Ml | 73 (84.9) | 96 (73.8) | 0.054 |
| Increased CK | 21 (24.4) | 13 (10.0) | 0.004** |
| Increased CK-MB | 41 (47.7) | 53 (40.8) | 0.316 |
| Increased LDH | 12 (14.0) | 8 (6.2%) | 0.0053 |
| Increased PCT | 33 (38.4) | 60 (46.2%) | 0.000*** |
| Death^#^ | 32 (37.2) | 13 (10.0%) | 0.000*** |

^#^ Causes of death were acute pulmonary edema, brainstem encephalitis and circulatory failure.

HFMD: hand, foot, and mouth disease, EV71: enterovirus 71, WBC: White blood cell, NT-proBNP: N-terminal of the prohormone brain natriuretic peptide, CK: Creatine kinase isoenzyme, CK-MB: Creatine kinase isoenzymeMB, LDH: Lactate dehydrogenase, PCT*:* procalcitonin, IVIG: Intravenous immunoglobulins.

**P* ＜0.05, ***P* ＜0.01,****P* ＜0.001
